# Design of Novel Auxetic Bi-Materials Using Convolutional Neural Networks

**DOI:** 10.3390/ma18081772

**Published:** 2025-04-13

**Authors:** Iulian Constantin Coropețchi, Dan Mihai Constantinescu, Alexandru Vasile, Andrei Ioan Indreș, Ștefan Sorohan

**Affiliations:** 1 Department of Strength of Materials, National University for Science and Technology POLITEHNICA Bucharest, Splaiul Independeței 313, 060042 Bucharest, Romania; iulian.coropetchi@mta.ro (I.C.C.); alexandru.vasile@mta.ro (A.V.); andrei.indres@mta.ro (A.I.I.); 2 Faculty of Aircraft and Military Vehicles, Military Technical Academy “Ferdinand I”, George Coșbuc Boulevard 39-49, 050141 Bucharest, Romania; stefan.sorohan@upb.ro; 3 Institute of Solid Mechanics of the Romanian Academy, Str. Constantin Mille No. 15, 010141 Bucharest, Romania

**Keywords:** convolutional neural networks, bi-material microstructures, auxetic materials, deep learning, microstructure optimization, greedy algorithm

## Abstract

A convolutional neural network (CNN) was developed to predict the Poisson’s ratio of representative volume elements (RVEs) composed of a bi-material system with soft and hard phases. The CNN was trained on a dataset of binary microstructure configurations, learning to approximate the effective Poisson’s ratio based on spatial material distribution. Once trained, the network was integrated into a greedy optimization algorithm to identify microstructures with auxetic behavior. The algorithm iteratively modified material arrangements, leveraging the CNN’s rapid inference to explore and refine configurations efficiently. The results demonstrate the feasibility of using deep learning for microstructure evaluation and optimization, offering a computationally efficient alternative to traditional finite element simulations. This approach provides a promising tool for the design of advanced metamaterials with tailored mechanical properties.

## 1. Introduction

Structural optimization is a major area of interest in mechanical engineering and has been continuously evolving for many years. Traditionally, it is used to minimize material mass and total deformation energy while maintaining mechanical strength or stiffness [1]. Structural optimization can be categorized into three main types: dimensional optimization, which aims to determine the optimal distribution of thickness in plates or cross-sectional areas in trusses; shape optimization, which focuses on identifying the best geometric configuration for a given domain; and topology optimization, which differs from the previous two by allowing material to be freely distributed within the design space [1].

In recent years, structural optimization has expanded beyond these classical approaches, encompassing topics such as cellular microstructures [2,3,4,5], compliant mechanisms [6], spinodal structures [7,8], and multiphysics problems [5,6,9]. Alongside these new applications, significant attention has been given to the mathematical methods used for solving optimization problems, which can be broadly classified into three main categories: gradient-based methods [10,11,12,13,14], direct search algorithms [15,16,17,18,19], and artificial intelligence-based algorithms, including deep learning (DL) [20,21,22,23] and machine learning (ML) [24,25,26,27,28]. Unlike traditional gradient-based methods, ML techniques can learn complex relationships from data and identify meaningful patterns without requiring explicit knowledge of the objective function’s gradient. These methods explore the solution space using learning-based algorithms that iteratively refine their predictions based on feedback, making them well suited for problems with large and complex search spaces.

On the other hand, auxetic materials offer significant advantages in applications, from increased toughness to impact resistance and energy absorption. When subjected to violent impacts, these materials become denser in the strike zone, thereby allowing for higher energy absorption, which enhances their protective capabilities. This property is particularly valuable in the design of advanced armor systems. Such outcomes can benefit from the use of ML strategies as developing auxetic structures [29,30], sandwich structures with lattice cores [31,32], FDM-printed auxetic configurations [33], and developing metamaterials [34,35], proving that ML optimizes their auxetic performance.

In mechanical engineering and optimization, the development of efficient methodologies for solving complex design problems remains an active and evolving field [2]. The increasing complexity of modern structures necessitates advanced optimization techniques capable of thoroughly exploring the design space and identifying configurations that meet performance criteria. With advancements in computing power and numerical methods, researchers now have access to a diverse range of optimization approaches, each with its own advantages and trade-offs.

This article explores the application of ML techniques in structural optimization, highlighting their flexibility, adaptability, and efficiency in solving a wide range of problems. The engineering application for metamaterial design uses a convolutional neural network together with the greedy algorithm, which leads to the development of bi-materials with negative Poisson’s ratios made from a hard and a soft material intended to be fabricated through additive manufacturing.

## 2. Problem Definition

**Figure 1:**
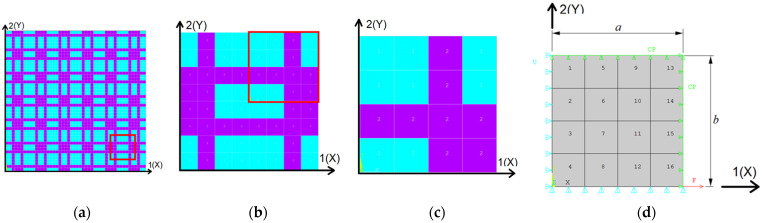
Description of the two-dimensional periodic domain formed by 2 materials with different properties: (**a**) portion of the periodic two-dimensional model marked with red square; (**b**) representative volume element (RVE) with double symmetry; (**c**) a quarter of the RVE used in analysis; (**d**) boundary conditions and dimensions.

To begin, we defined an optimization problem for a composite material, which serves as a test scenario for the analyzed method. The selected optimization problem, adapted from [2] with certain modifications, involved a two-dimensional periodic domain composed of two distinct materials, as illustrated in Figure 1a. The materials had known elastic moduli, *E*_1_ and *E*_2_, as well as Poisson’s ratios, *ν*_1_ and *ν*_2_. The harder material is represented in magenta, while the softer material appears in cyan. No constraints were imposed on the proportion of each material in the domain, ensuring that the search space remained as unrestricted as possible. The objective of this optimization problem was to determine the optimal material distribution within the domain that minimizes the effective Poisson’s ratios in two orthogonal directions.

A representative unit cell was identified within the given domain, as shown in Figure 1b. Due to symmetry considerations, only a quarter of this unit cell needed to be analyzed, as depicted in Figure 1c. This quarter-cell, with overall dimensions *a* × *b* (where *a* = *b* = 1 mm for this specific case), was studied under appropriate boundary conditions (supports and loads), as illustrated in Figure 1d. The domain was meshed using only 16 equal quadrilateral finite elements. To simulate the behavior of this quarter-cell in the periodic domain, symmetry boundary conditions were applied: displacement symmetry along the *X*-axis on the edge *X* = 0 and along the *Y*-axis on the edge *Y* = 0. Additionally, displacement coupling was enforced along the *X*-axis on the edge *X* = *a* and along the *Y*-axis on the edge *Y* = *b*. These boundary conditions ensured that the analysis of the quarter-cell was equivalent to performing computations on the entire periodic domain.

The finite elements used in the discretization can be quadrilateral elements with either four or eight nodes. In a linear elastic analysis, stress distribution within each finite element varies unless a reduced integration quadrilateral element or stress averaging is employed. However, for computational simplicity, constant stress values are typically assumed for each finite element in practical applications.

According to [36], determining the elastic material constants for the two-dimensional case requires three distinct loading conditions, which can be applied to the quarter-cell of the RVE under the boundary conditions specified in Table 1. As a remark, Sym means symmetric, and ASym means asymmetric.

The specific strains imposed arbitrarily for the three load cases can be chosen, for example, as $\bar{\varepsilon_{01}}=\bar{\varepsilon_{02}}=\bar{\gamma_{03}}=0.1$. These strains correspond to applied displacements, as detailed in Table 1, where the resulting nonzero reactions represent the necessary forces. For the same three loading cases, Table 2 presents the calculation formulas for the effective elastic constants of the material.

For the given problem, a key parameter of the representative unit cell is the effective Poisson’s ratio, which can be defined by the following equation:

(1) $$\nu_{12}=-\frac{{\bar{\varepsilon }}_{y}}{{\bar{\varepsilon }}_{x}}=-\frac{\frac{1}{V}\sum_{i=1}^{NE}V_{i}\varepsilon_{yi}}{\frac{1}{V}\sum_{i=1}^{NE}V_{i}\varepsilon_{xi}}$$

The terms in Table 1 and Equation (1) represent:

- *E*_1_—the effective (averaged) elastic moduli in direction 1 (*X*);
- *E*_2_—the effective (averaged) elastic moduli in direction 2 (*Y*);
- *G*_12_—the effective (averaged) transverse elastic moduli in direction 12 (*XY*);
- *ν*_12_—the Poisson’s coefficient when the material is subjected to tensile stress in direction 1 (*X*) and measured in direction 2 (*Y*);
- *ν*_21_—the Poisson’s coefficient when the material is subjected to tensile stress in direction 2 (*Y*) and measured in direction 1 (*X*);
- $\bar{\sigma }$—the averaged stress on the entire domain;
- $\bar{\varepsilon }$—the averaged strain on the entire domain;
- *V*—the volume of the entire domain;
- *NE*—the number of elements;
- *σ_i_*—the mean stress in element *i*;
- *ε_i_*—the mean strain in element *i*;
- $\bar{\tau_{xy}}$—the averaged shear stress on the entire domain.

Equation (1) is an adaptation of the formula presented in [2], incorporating the finite element volume into the calculation. When the mesh was uniform, the finite element volume could be omitted, as the contribution of each element to stress or strain components remained proportional. To evaluate a given material configuration, two loading cases were applied to determine Poisson’s ratios in the two orthogonal directions—specifically, tension along the X-axis and tension along the Y-axis.

As detailed in the following sections, the optimization problem’s objective function required only the first two loading cases. However, by also considering the third case, valuable insights could be gained regarding the behavior of composite cellular structures.

For the optimization problem defined in this report, the two materials were assigned the following mechanical properties: the soft material had a Young’s modulus of *E*_1_ = 1 MPa and a Poisson’s ratio of *ν*_1_ = 0.4, while the hard material had a Young’s modulus of *E*_2_ = 100 MPa and a Poisson’s ratio of *ν*_2_ = 0.2. This resulted in a stiffness contrast of 100:1 between the two materials.

Such a ratio between Young’s moduli is commonly observed in various engineering materials. For example, PETG, with a Young’s modulus around 1939 MPa [37], is much stiffer than TPU 85A [38], which has a Young’s modulus of about 20 MPa, resulting in a ratio of 100. Similarly, a common used steel like S235JR, with a Young’s modulus around 210 GPa [39], is significantly stiffer than polycarbonate (PC), which has a modulus of about 2.3 GPa [40], also yielding a ratio close to 100. These differences illustrate how materials with widely varying moduli can be used for different applications, where the stiffer materials are better suited for load-bearing structural components, while the more flexible ones are ideal for applications requiring elasticity and impact resistance.

To validate the performance of an optimization algorithm, it is essential to determine the optimal solution. This allows for an assessment based on the time required to reach the solution or, if the optimum is not achieved, how closely the result approximates the best possible value. The optimal solution is identified by exhaustively evaluating all possible distributions of the two materials in the previously described problem, which consists of 16 finite elements. This approach ensures that no potential configuration is overlooked. In the scientific literature, this exhaustive evaluation method is commonly referred to as brute force [25].

To evaluate all possible distributions of the two materials, it is first necessary to determine the computational effort involved. In the absence of any constraints on the proportions of the two materials, the total number of possible configurations can be calculated using the following equation:

(2) $$n_{comb}=m^{n}$$

In Equation (2), the terms represent:

- *n_comb_*—the total number of combinations;
- *m*—the number of materials used;
- *n*—the number of finite elements.

**Figure 2:**
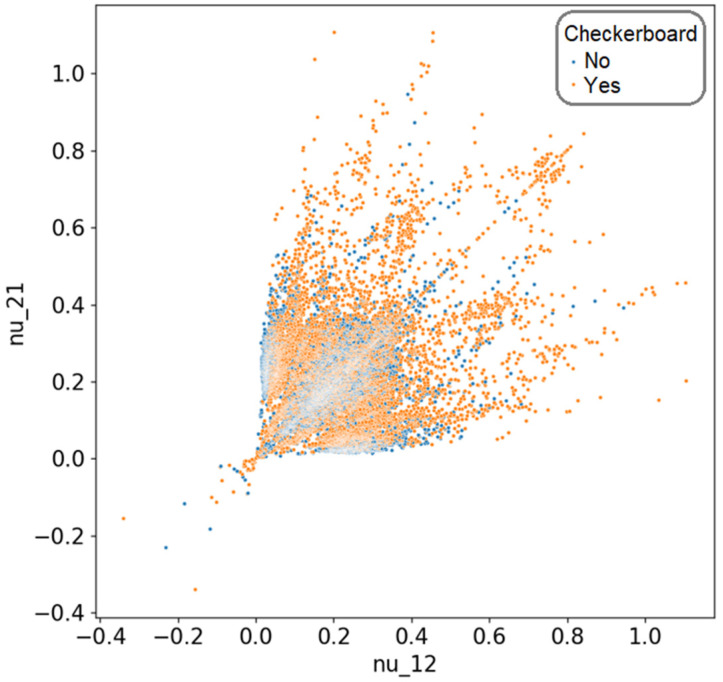
The distribution of all solutions based on the value of Poisson’s coefficients, differentiated by the presence of elements in a checkerboard pattern.

In this case, for *m* = 2 and *n* = 16, we obtained a total of 65,536 possible combinations. Given that the goal was to identify the distributions with the lowest effective Poisson’s ratio values, these solutions could be visualized as a scatter plot, where *ν*_12_ is represented on the horizontal axis and *ν*_21_ on the vertical axis. The complete set of solutions for this scenario is illustrated in Figure 2.

**Figure 3:**
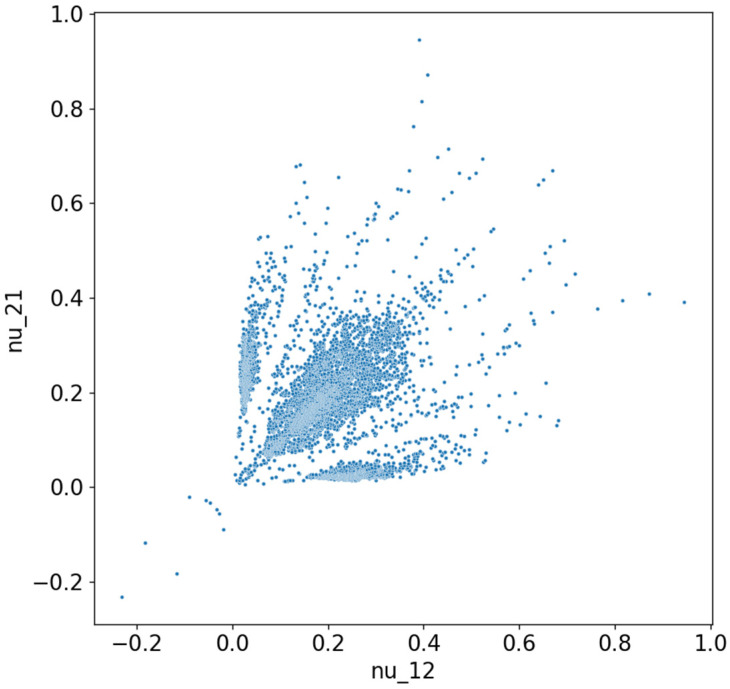
The distribution of all solutions that do not contain elements in a checkerboard pattern, based on the value of Poisson’s coefficients.

A convergence study was conducted on this type of problem [41], leading to the conclusion that, in order to eliminate computational errors arising in finite element calculations, it was necessary to implement a mesh with nine equally sized finite elements per material domain. Additionally, it was demonstrated that solutions featuring checkerboard-like element distributions were significantly more affected by the mesh resolution, with their performance decreasing as the number of finite elements per material domain increases. Checkerboard elements refer to material elements that are connected to other elements of the same type only through a single node, rather than along an entire edge, as would be the case with QUAD8-type elements. Considering these findings, this article adopted a mesh approach using a single finite element per material domain while excluding solutions that exhibited checkerboard-connected elements. For the given problem, an analysis of the solutions obtained through brute-force evaluation revealed that 23,858 solutions did not contain checkerboard elements, accounting for approximately 36.4% of the total 65,536 possible solutions. A graphical representation of these valid solutions can be found in Figure 3.

**Figure 4:**
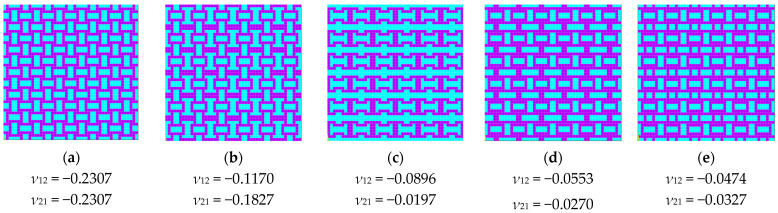
The periodic representation of solutions with a negative Poisson’s coefficient value for the 4 × 4 model: (**a**) 50% hard material; (**b**) 50% hard material; (**c**) 43.75% hard material; (**d**) 56.25% hard material; (**e**) 56.25% hard material.

If we analyze the previous figure, we can observe that it is symmetric with respect to the first bisector. This symmetry was expected in this representation, as certain solutions correspond to others when rotated by 90°. Since the two loading cases also corresponded to a 90° rotation, solutions with identical material domain distributions—rotated versions of each other—appeared mirrored in the plot. In the bottom-left corner, we find solutions with a negative Poisson’s ratio, totaling 34 configurations, out of which only 5 are unique (independent) periodic structures. These represent less than 0.15% of the entire solution space. The reason why all 34 points are not distinctly visible in the figure is that some solutions overlap due to identical values of the two Poisson’s ratio components. The independent periodic solutions that exhibited a negative Poisson’s ratio are shown in Figure 4.

To analyze the differences between the 4 × 4 configurations with the same percentage of hard material, we must consider both their geometric arrangement and how material distribution influences their auxetic behavior. Among the five solutions obtained, two configurations contained 50% hard material, while two others had 56.25% hard material. Despite having the same material proportion, the spatial distribution of the hard material significantly influenced their mechanical properties. The key differences came from the connectivity between hard material regions, which affected how the structure deformed under load and the placement and shape of soft material zones, which influenced the flexibility and Poisson’s ratio variation. Additionally, the configuration with 43.75% hard material provided a useful reference, as it demonstrated that a lower percentage of hard material can still achieve auxetic behavior, possibly due to an optimized topology that enhances deformation mechanisms.

Moving to the next step, for a similar configuration with 36 material elements in the domain—corresponding to a 6 × 6 grid—the total number of possible configurations was 2^36^, which exceeds 68 billion. The previously imposed condition that eliminated solutions featuring checkerboard-like material distributions became increasingly difficult to verify in this scenario. Even if the verification of a single solution took only a short time, the sheer number of possible configurations significantly increased the computational effort. To estimate the total number of solutions that did not contain checkerboard elements, a Monte Carlo simulation was performed. In this simulation, 100 sets of 100,000 randomly generated solutions were evaluated. The results showed that, on average, approximately 93.5% of the solutions contained checkerboard elements, leaving only 6.5% that did not. Based on this percentage, we estimated that for the 36-element configuration, the total number of solutions without checkerboard patterns was approximately 4.5 billion.

Even with this reduced number of valid solutions, the computational effort remained impractical. Given that a single finite element simulation for this configuration takes approximately 3 s, the total time required to evaluate all possible solutions would exceed 400 years on the available hardware. Thus, we can conclude that finding the optimal solution using a brute-force approach is not feasible in this case.

## 3. Model

### 3.1. Data Preparation

The workflow for artificial intelligence models involved several key stages. The first step for a user was preparing the dataset, which required creating a sufficiently large database to ensure proper training that met the model’s requirements. The way the dataset is constructed was also crucial, as it must provide diverse data covering the entire solution space while maintaining consistency in format. Once the training data were prepared, the next stage was model construction. Each network or model learned and performed effectively depending on its specific architecture. If a chosen network did not align well with the expected outcome, it was unlikely to achieve high performance. There was a strong interdependence between the training dataset, network architecture, and desired results—each factor influencing the others. The next phase involved training the models. With the dataset ready and the model constructed, the training process began. This step consisted of an automated adjustment of the network’s weights to improve its success rate. If training resulted in a satisfactory performance level, the model moved to the validation phase. During validation, the trained network was tested on datasets that were not used in training, ensuring it could achieve a high success rate even when presented with previously unseen data.

For the problem described in Section 2, the 4 × 4 variant had a total of 23,858 possible solutions, making it feasible to use all solutions as the input dataset. Since a brute-force approach was applied to the 4 × 4 case, allowing for the determination of all possible solutions, 90% of these data were allocated for training, while the remaining 10% were reserved for validation.

For the 6 × 6 variant, Section 2 determined that there were over 4.5 billion solutions without checkerboard-patterned elements, making a brute-force analysis impractical. In this case, it was necessary to establish an appropriate number of training samples. According to [42], learning theory explains how machine learning algorithms can generalize correctly from a limited training dataset, despite the logical contradiction of inferring general rules from finite data. Machine learning addresses this issue by generating probabilistic rules that are likely correct for most cases, rather than absolute logical rules. However, the “No Free Lunch” theorem [43] states that, on average, all classification algorithms perform equally poorly across all possible data distributions. The key to overcome this limitation is to make informed assumptions about the types of distributions encountered in real-world scenarios. The goal of machine learning research is not to develop a universal algorithm but to understand which data distributions are relevant for practical applications and design algorithms that perform well for those specific distributions.

**Figure 5:**
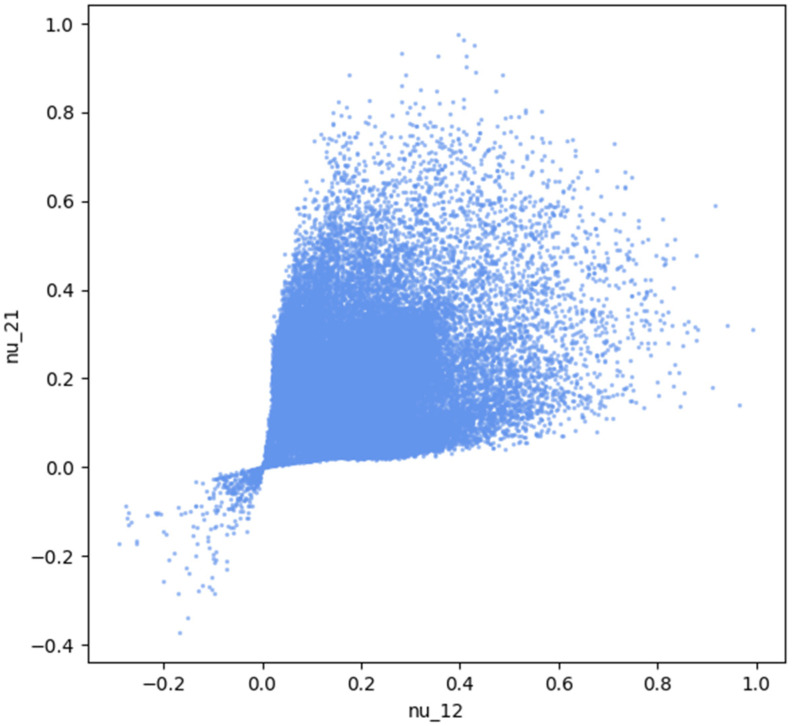
The distribution of all 70,000 solutions for the 6 × 6 model, based on the value of Poisson’s coefficients.

For the 6 × 6 configuration with 36 material elements per domain, finite element simulations have been used to determine 70,000 solutions. To check that the solutions sufficiently covered the entire solution space, the distributions of these solutions are presented in Figure 5. We can see that the solutions presented values for the Poisson’s coefficient starting from −0.4 up to 1.0. The following section presents the methodology for constructing neural networks and explains how the data from the 4 × 4 network could be leveraged to train networks for the 6 × 6 configuration.

During training, it was observed that when the input data provided to the network were presented as a 4 × 4 matrix with element values of 1 or 2, the training process became slower, and the performance was lower. This issue could be explained by the fact that the statistical model on which convolutional neural networks or artificial intelligence models are built generally works better with binary values of 0 and 1 rather than 1 and 2. Although the vectors defining a configuration were noted with binary values 1 and 2 for easier implementation in PyAnsys, it was necessary to transform these values into 0 and 1 to improve the process. As for the input data matrix in the 4 × 4 form, this representation corresponded to a quarter of the representative cell. While this was the simplest representation of the periodic model, to improve the training process, it was necessary to transform the 4 × 4 matrix into an 8 × 8 matrix, which represents a complete representative volume. The reason this transformation was necessary is related to the boundary conditions imposed for the finite element model, which the neural network could not efficiently detect on its own.

### 3.2. Model Construction

**Figure 6:**
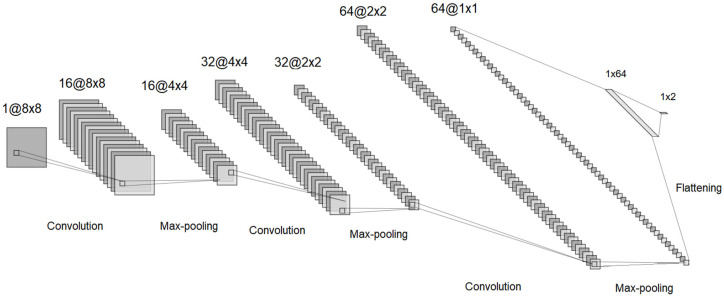
The convolutional neural network structure for the 4 × 4 configuration.

For the optimization process, it was proposed to use a convolutional neural network model that learns from the previously generated training data and can predict the mechanical properties of a given configuration. Based on the previously presented information, a network structure is proposed, which is shown in Figure 6.

Analyzing the figure, we observed a total of 9 layers, 7 of which were hidden layers. In the early stages of the network, there were three convolutional layers, each followed by a max-pooling layer. The convolutional layers used 3 × 3 kernels and an increasingly larger number of filters (starting from 16, then 32, and finally 64). This progressive increase in filters ensured that the model effectively captured the important features needed for learning the objective function. The small kernel size of 3 × 3 was chosen to extract fine-grained spatial details while maintaining computational efficiency. The max-pooling layers, intercalated between the convolutional layers, played a crucial role in gradually reducing the spatial dimensions of the input. This reduction helped to focus on the most relevant features while also decreasing computational complexity and preventing overfitting. After the feature extraction process, the network included a flattening layer that converted the 2D feature maps into a 1D vector, preparing it for the final fully connected layers (dense layers). The final dense layer transitioned from 64 neurons to 2 output variables are representing the Poisson coefficients for the two loading directions. These values served as the model’s final predictions, indicating how the composite material would behave under different applied loads.

The first layer was the input layer, where a binary matrix corresponding to a representative cell configuration of the composite material was provided. In this first layer, 16 convolutional filters were applied, resulting in 16 matrices, which then passed through a max-pooling layer that reduced the matrix dimensions from 12 × 12 to 6 × 6. The process continued with additional convolutional layers intercalated with max-pooling layers, where more filters were applied, thus increasing the number of intermediate matrices, while at the same time reducing their dimensions. The second to last layer, the flattening layer, combined the 64 matrices of size 2 × 2 into a value vector, which was then passed through a fully connected layer and reduced to 2 values corresponding to the Poisson coefficients for the two loading directions.

**Figure 7:**
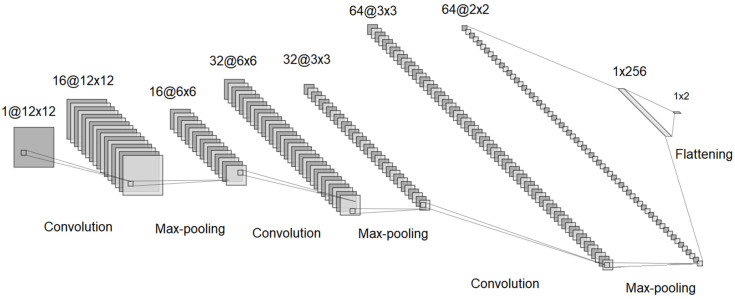
The convolutional neural network structure for the 6 × 6 configuration.

Regarding the convolutional neural network for the 6 × 6 configuration, the transfer learning technique was employed, which involved transferring the weights determined after training the network in the 4 × 4 configuration to the 6 × 6 network at the beginning of the training process. Transfer learning [44] aids the training process by reducing the number of required training data and epochs. Essentially, using the convolutional layer weights determined by training the 4 × 4 neural network in the 6 × 6 network’s convolutional layers allowed the training process for the 6 × 6 network to not start from scratch. Thus, the structure of the 6 × 6 network was similar to that of the 4 × 4 network and is shown in Figure 7.

As mentioned earlier, it can be observed that the structure of the 6 × 6 network was similar to that of the 4 × 4 network, but it differed in the size of the input and output data for each layer.

### 3.3. Model Training

According to the classification of artificial intelligence models [43], convolutional neural networks fall under supervised learning models. Assuming there is a training dataset of input–output pairs, where the output data are the result of passing the input data through a function f, the goal of supervised learning, as stated in [44], is to find a function h that approximates the true function f as closely as possible. Since the network structure has already been defined, the structure of the function h to be determined is also defined, and through training, the weights that define this function are determined. In our case, since the output data, defined by the Poisson coefficients, were real variables, the problem was a regression problem.

Supervised learning involves exploring the function space to find the function that best fits the relationship between the provided input and output data used for training. The learning process is iterative, taking place over multiple training epochs, and uses an optimizer to adjust the function’s weights throughout the learning process. In this case, the Adam optimizer [45] was used due to its high performance compared to other models. In machine learning, especially in deep learning, an epoch refers to a complete pass through the entire training dataset during the training process. In each epoch, the model processes all training samples, adjusting its parameters (weights) through backpropagation to minimize the error. Typically, training requires several epochs for the model to gradually improve its performance by learning patterns from the data. However, too few epochs may result in underfitting (where the model fails to learn sufficient features), while too many epochs may lead to overfitting (where the model memorizes the training data but fails to generalize to new data).

To track the learning performance throughout the training process, a function was used to quantify the loss the model has at each training step. There are many loss functions with varying performance depending on the type of problem. The literature [44] suggests several functions for quantifying loss, but for a regression problem, the mean squared error is recommended. This error represents the sum of the squared differences between the predicted values and the actual values, divided by the number of points. The formula for calculating the mean squared error is presented in Equation (3):

(3) $$MSE=\frac{1}{n}\sum_{i=1}^{n}{\left(y_{i}-{\hat{y}}_{i}\right)}^{2}$$

In Equation (3), the terms represent:

- *MSE*—the mean squared error;
- *n*—the number of points;
- *y_i_*—the real value of the objective function for point *i*;
- $\hat{y_{i}}$—the predicted value of the objective function for point *i*.

**Figure 8:**
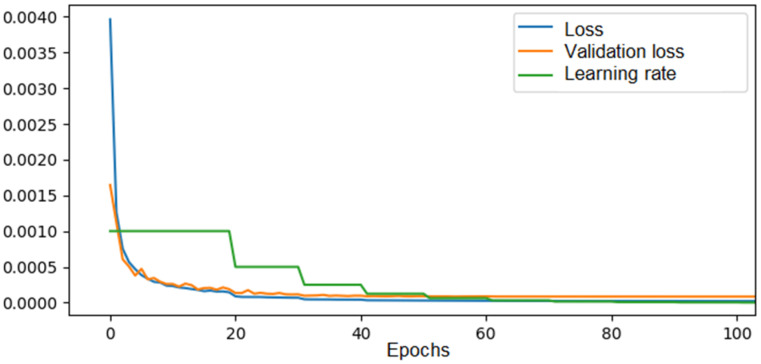
The loss, validation loss, and learning rate evolution across epochs during training of the CNN in the 4 × 4 configuration.

For the convolutional neural network model for the 4 × 4 configuration, training was performed on a dataset consisting of 90% of the total solutions obtained via brute force over 100 epochs with a decreasing learning rate, dependent on the mean squared error, starting at 0.1. The evolution of the loss for both the training and validation data can be seen in Figure 8.

As we can observe, both the training error and the validation error gradually decreased throughout the learning process until they reached a plateau after approximately 60 epochs, with values of 0.00002 for the training error and 0.00008 for the validation error. The learning rate followed a halving function, where if there were no significant improvements in the validation error after 10 epochs, it was halved until it reached the imposed minimum.

**Figure 9:**
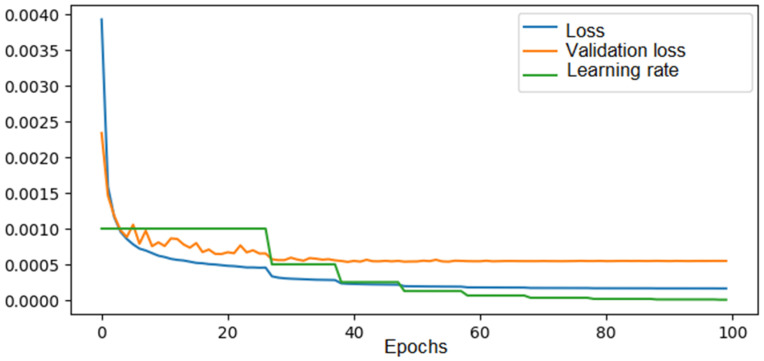
The loss, validation loss, and learning rate evolution across epochs during training of the CNN in the 6 × 6 configuration—70,000 solutions in the training set.

Regarding the 6 × 6 model, an initial training was conducted using a dataset of 70,000 solutions for 100 epochs of learning, and the evolution of the mean squared error was analyzed in this case. The evolution of the loss function seen in Figure 9.

Similar to the training process of the network for the 4 × 4 configuration, the error values decreased over the epochs. However, in this case, there was a greater difference between the validation error and the training error, with values reaching 0.00055 and 0.00018, respectively. This effect arose because, for the 8 × 8 configuration, training was done on a dataset containing all the known distributions for that configuration. In this case, a dataset of 70,000 solutions, compared to the total possible solutions determined in Section 2 (4.5 billion solutions), represents training with just 0.00015% of the total solutions.

**Figure 10:**
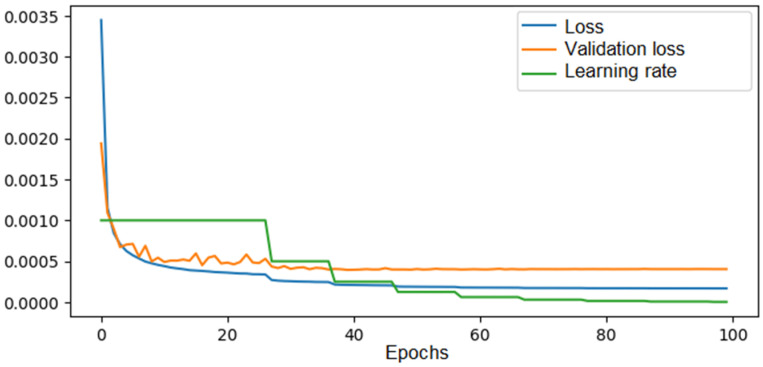
The loss, validation loss, and learning rate evolution across epochs during training of the CNN in the 6 × 6 configuration—140,000 solutions in the training set with data augmentation.

A commonly used technique to improve the training dataset is data augmentation. For example, in our case, a solution rotated by 90° corresponded to another solution in the entire solution space, but with the values of the Poisson coefficients swapped. By applying this procedure to the entire dataset, we obtained a dataset consisting of 140,000 solutions, or 0.0003% of the total solutions. In this way, the training loss decreased to 0.00016, a reduction of approximately 11%, and the validation loss decreased to 0.0004, a reduction of approximately 27%. The results are shown in Figure 10. Similarly, the procedure of symmetry across the horizontal axis was applied to the entire dataset, and with a set of 240,000 solutions, a validation error of 0.00035 was achieved.

**Figure 11:**
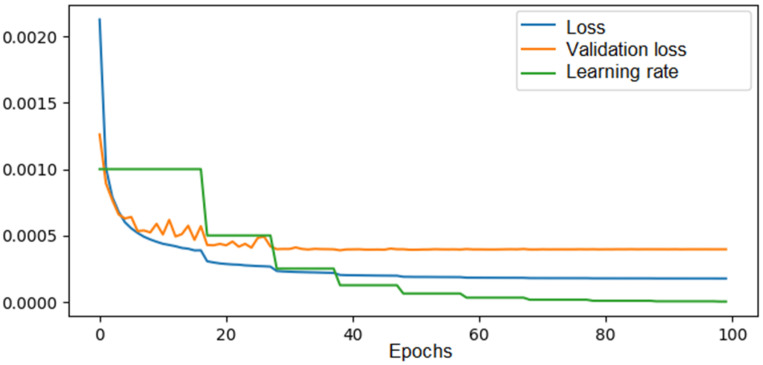
The loss, validation loss, and learning rate evolution across epochs during training of the CNN in the 6 × 6 configuration—140,000 solutions in the training set with data augmentation and transfer learning.

In another case, transfer learning was applied from the trained 4 × 4 model to an untrained 6 × 6 model. This process involves taking the weights of the similar layers from the trained network and transferring them to the corresponding layers of the network that needs to be trained. The results regarding the loss evolution are presented in Figure 11.

From the figure, we can observe the impact of transfer learning from the 4 × 4 network to the 6 × 6 network on the initial learning performance. In the previous case, the training loss started at approximately 0.0035, whereas in this case, it began at a lower value of 0.0022. Similarly, the validation loss, which initially started at 0.002 in the previous case, began at 0.0013. This suggests that some of the features learned by the trained 4 × 4 network could be retained to a certain extent in the 6 × 6 configuration, potentially reducing the required training dataset size or the number of training epochs. At the same time, we noticed that the final training and validation losses did not differ significantly, leading to the conclusion that transfer learning could be effectively used in certain cases to reduce the training time for very large networks. This section is summarized in a comparative way into Table 3.

### 3.4. Model Validation

Earlier, we discussed the data preparation process and the training method for the convolutional network. Regarding network validation, when dealing with categorical variables, an accuracy or success rate can be defined as the percentage of cases where the network correctly classifies the input data into the corresponding category. However, in our case, the results were not categorical but instead referred to the two numerical values of Poisson’s ratios.

**Figure 12:**
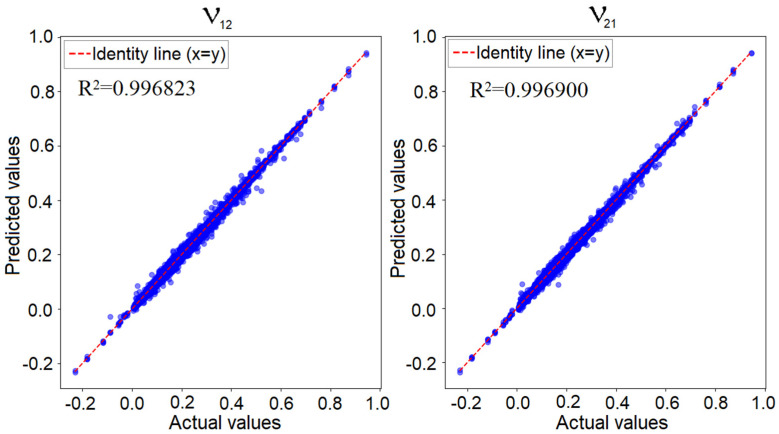
Predicted values versus actual values for the convolutional network in the 4 × 4 configuration—Poisson’s ratio along the X-axis (**left**) and along the Y-axis (**right**).

Figure 12 illustrates the predicted values versus the known actual values for the convolutional neural network applied to the 4 × 4 model, focusing on the two Poisson’s ratio values. If the network’s prediction error was zero, all points would align perfectly along the identity line, representing the region where the predicted values matched the actual values. In reality, as previously shown, the minimum training loss was 0.00002, while the validation loss was 0.00008. This indicates that while predictions were not entirely identical to the true values, the error was sufficiently low to confirm the network’s performance. In such a graphical representation, the closer the points are to the identity line, the better the network’s predictive accuracy is.

**Figure 13:**
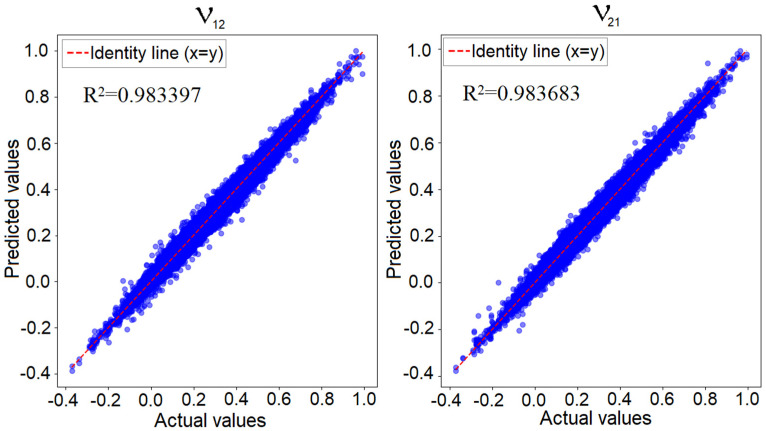
Predicted values versus actual values for the convolutional network in the 6 × 6 configuration—Poisson’s ratio along the X-axis (**left**) and along the Y-axis (**right**).

Regarding the 6 × 6 model, a similar representation for the best-trained network can be observed in Figure 13. As previously mentioned, the losses obtained for this network were higher than those for the 4 × 4 model. This was due to the significantly larger number of possible configurations compared to the training dataset, which limited the network’s ability to fully capture all configuration characteristics. However, the figure shows that the points were closely aligned with the identity line (*y* = *x*), indicating strong predictive performance. As noted in the specialized literature [44], there is no single error threshold that definitively validates a model. The validation of a neural network always depends on the user’s requirements. In this case, given the reported error values relative to the two Poisson’s ratios, we can conclude that the network was effectively validated.

## 4. Results and Discussion

The convolutional neural network model has proven to be robust, exhibiting a very low prediction error. This suggests that the training was performed with a sufficiently large dataset, even though it represented only 0.0006% of the entire solution space. Additionally, it indicates that the training parameters were well optimized, contributing to the model’s strong performance.

Ultimately, we obtained a model capable of accurately predicting the mechanical properties of a given configuration without performing complex calculations similar to finite element analysis. By leveraging this model, we could apply direct search methods to identify the optimal solution within the solution space.

Based on previous research [46], it was established that the greedy algorithms performed well in solving these types of problems due to their fast convergence and computational efficiency, requiring far fewer objective function evaluations compared to methods like Genetic Algorithms (GA) and Particle Swarm Optimization (PSO). While greedy algorithms may sometimes yield suboptimal solutions, they excel in avoiding deadlocks and escaping local minima, particularly in larger problem instances (6 × 6 grids), where GA and PSO tend to struggle. In previous studies, greedy algorithms have been found to be the optimal or near-optimal solution three out of five times while requiring just 256 evaluations, whereas GA needed over 5000. This makes it highly suitable for large-scale problems where computational resources are limited. Although methods like PSO and Simulated Annealing (SA) achieve better solution quality in smaller cases, their higher computational costs make greedy algorithms a more practical choice when speed and efficiency are prioritized.

**Figure 14:**
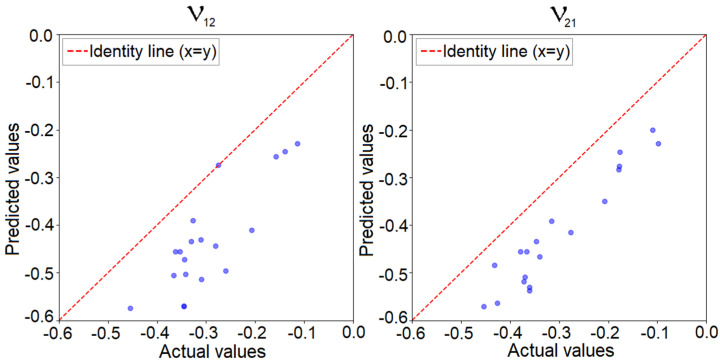
Predicted versus actual values of Poisson’s ratio for auxetic solutions determined using the greedy algorithm for the 6 × 6 model.

In this regard, we applied the algorithm along with the trained convolutional neural network for the 6 × 6 model. Figure 14 presents the predicted values of Poisson’s coefficients from the neural network compared to the actual values verified through finite element analysis for 20 auxetic solutions determined by the algorithm. To enhance the performance of the greedy algorithm, during each iteration, it evaluated all configurations obtained by modifying one element from the domain, as well as all configurations derived by modifying two elements. At each iteration, the algorithm evaluated up to 1296 solutions, excluding those that contained elements connected in a checkerboard pattern, as we established those to be eliminated due to errors.

**Figure 15:**
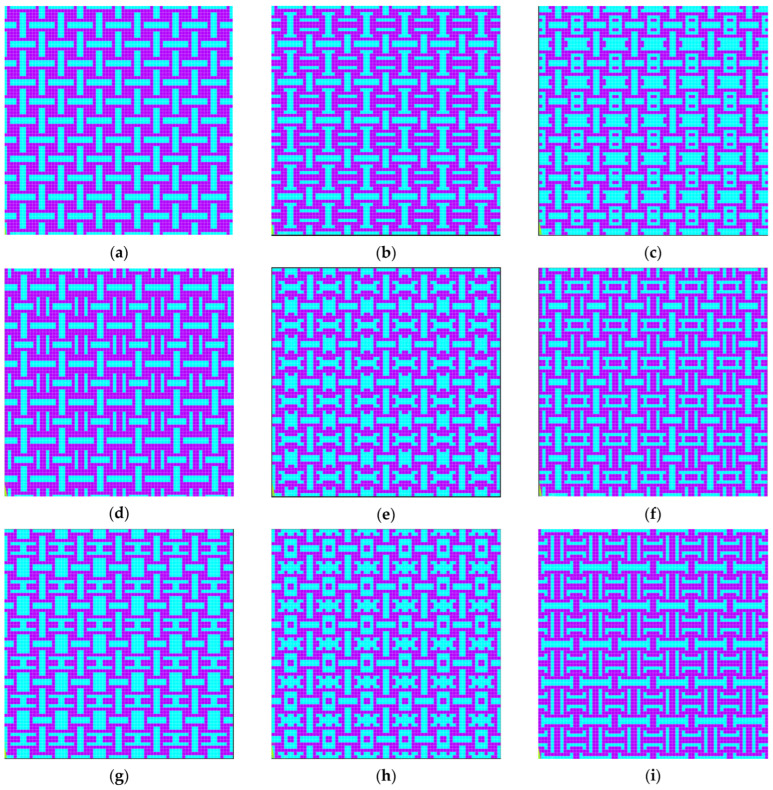
The top nine solutions determined by the greedy algorithm combined with the convolutional neural network for the 6 × 6 model: (**a**) 56.56% hard material; (**b**) 58.33% hard material; (**c**) 47.22% hard material; (**d**) 61.11% hard material; (**e**) 52.78% hard material; (**f**) 58.33% hard material; (**g**) 50.00% hard material; (**h**) 50.00% hard material; (**i**) 61.11% hard material.

We can observe that, compared to the previously presented results where a similar representation was made for the solutions used for training and validation, for these solutions, the predicted value was much lower than the actual value determined through finite element analysis, with the error reaching up to 133%. This can be explained by the fact that in the training dataset, which consisted of 280,000 solutions, only about 1500 solutions were auxetic, totaling roughly 0.53%, and these solutions presented negative values for Poisson’s coefficient, with the smallest value encountered being −0.371. Thus, although the network had learned to predict Poisson’s coefficient for randomly selected solutions, it exhibited a larger error for the extremes of the solution space. At the same time, even though the actual value significantly differed from the predicted value, the solutions determined were still auxetic, and the order of the predicted coefficients matched the order of the real coefficients. To improve the accuracy of the model for auxetic configurations, more auxetic solutions have to be added to the dataset for better generalization of the CNN in the area of auxetic configurations of the solution space. Figure 15 presents the best 9 solutions determined by the algorithm in the periodic representation of the structure.

In terms of computational efficiency, a direct comparison can be made based on the time required to evaluate solutions. Finite element method simulations, which serve as the ground truth for evaluating auxetic behavior, take approximately 3 s per solution. A greedy algorithm, which systematically searches for improved solutions, evaluates a maximum of 1296 solutions per iteration. To obtain the nine best solutions presented, 50 independent runs of the greedy algorithm were performed, with an average of eight iterations per run. This means that a total of approximately 518,400 function evaluations were conducted using the greedy approach combined with trained CNN predictions.

In contrast, the CNN significantly improved computational efficiency. The trained model was capable of evaluating 280,000 solutions in just 13.3 s. This highlights the advantage of CNNs in terms of rapid inference, as once trained, the model can produce near-instantaneous predictions without requiring costly FEM evaluations. Given that the greedy algorithm relies on multiple iterations of FEM simulations, its computational burden is significantly higher, making CNNs a more practical alternative for large-scale design exploration.

**Figure 16:**
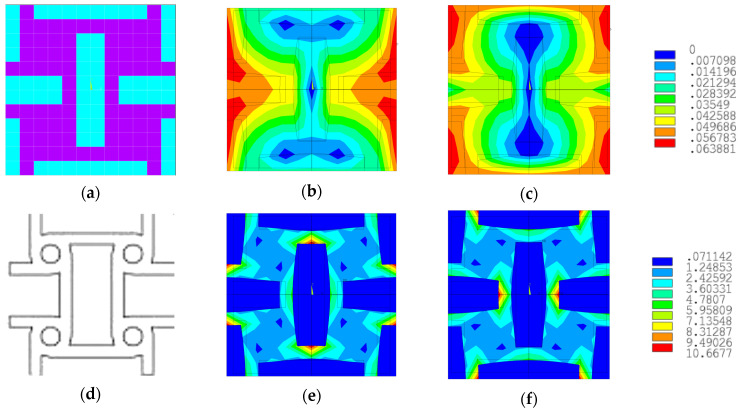
The solution with the lowest Poisson’s ratio, the displacement and stress fields for the two loading cases, and its similarity to the tetra-anti-chiral cell: (**a**) representative volume element; (**b**) displacement field axial loading on X-axis; (**c**) displacement field axial loading on Y-axis; (**d**) tetra-anti-chiral cell [47]; (**e**) stress field axial loading on X-axis; (**f**) stress field axial loading on Y-axis.

We can observe in the previously presented figure that all solutions shared a common element, namely the presence of a soft material rectangle that alternated orientation along the X (horizontal) or Y (vertical) direction in the periodic structure. The solution with the lowest Poisson’s coefficient values is the one in Figure 15a, where the Poisson’s coefficient had equal values of −0.4543 in both orthogonal directions. Analyzing the literature, we can see that this solution was very similar to the tetra-anti-chiral cell presented in Figure 16d.

In Figure 16, the representative cell for the best solution obtained is shown in (a), and the displacement field for axial loading along the X-axis is shown in (b) and along the Y-axis in (c). Additionally, the equivalent stresses in the representative cell for axial loading along the X-axis (e) and Y-axis (f) are displayed. The displacement field and equivalent stresses were obtained through finite element analysis using the loading cases presented in Section 2, specifically tension along the X-axis and tension along the Y-axis.

## 5. Conclusions

This research presents optimization models based on artificial intelligence techniques for composite materials with representative volume elements, aiming at identifying metamaterials with auxetic behavior. The methodology combines traditional optimization techniques, such as direct search methods, with advanced learning models to efficiently determine optimal solutions.

The application of convolutional neural networks for predicting the mechanical properties of representative volumes has proven to be highly efficient. By training the network with a dataset consisting of configurations of representative volumes and the values of Poisson’s coefficients, representing only 0.006% of the entire solution space, it was able to accurately estimate the mechanical properties of the configurations. Although the model exhibited a higher prediction error for the Poisson’s coefficients at the extremes of the solution space, this has been explained by the nature of the training dataset. The low prediction error of the convolutional neural network highlights the potential of machine learning models in addressing the challenges posed by computationally expensive simulations.

By integrating the convolutional neural network after completing the training process with the greedy algorithm, the optimization process for determining auxetic materials was streamlined. The greedy algorithm navigated relatively easily through the solution space to determine high-performance configurations, a process that was facilitated by the reduced computational time provided by the trained network compared to traditional finite element analysis.

The computational efficiency of the proposed CNN-based method can be analyzed by considering the time required for different stages of the process. First, the dataset was generated by evaluating 70,000 randomly generated solutions using finite element simulations, with each evaluation taking approximately 3 s, leading to a total time of around 58 h. Training the CNN on this dataset required approximately 36 min, after which the trained model could predict Poisson’s ratio values for a given structure almost instantly. However, to ensure high-quality solutions, predictions were further refined using a greedy algorithm with checkerboard constraints, which required around 30 min per 50 greedy evaluations, with each greedy optimization performing multiple iterations. This demonstrates that, while finite element simulations were computationally expensive, once trained, the CNN significantly reduced evaluation time, allowing for a much more efficient search for optimal auxetic configurations. If the greedy evaluations were performed using FEM simulations instead of CNN prediction, the total computational time would have been around 432 h instead of 59 h considering the whole process of generating solutions, training the model, and performing greedy evaluations.

The CNN-based method was designed to efficiently handle relatively small systems, such as the 6 × 6 grid configuration. However, extending this approach to larger or more complex systems presents several challenges. One potential issue is the increased computational complexity as the size of the problem grows. Larger configurations, such as 8 × 8 grids or more complex material layouts, would require significantly more data points for training to capture the diverse range of possible configurations, leading to longer training times and potentially higher memory usage.

Another challenge is the resolution of the problem space. As the grid size increased, the complexity of the patterns and features within the material structure increased as well. One way to handle this is by incorporating more convolutional layers into the CNN, which can capture patterns at different levels of granularity, improving the model’s performance.

The optimal solution determined by the greedy algorithm combined with the trained convolutional neural network showed a high degree of similarity with known cells in the literature, referred to as tetra-anti-chiral. This similarity demonstrated the validity and capability of the optimization model to successfully navigate the solution space. At the same time, the emergence of such a structure highlighted that the convolutional neural network model had “learned” and effectively utilized the mechanical principles underlying auxetic behavior. The appearance of a solution similar to tetra-anti-chiral cells indicates that these solutions were not only theoretically robust but also naturally emerged from data-driven optimization processes, further validating their relevance in the field of metamaterials.

## Figures and Tables

**Table 1:** Boundary conditions impose for the 3 load cases [36].

Load Case	Nodes on Line	Displacement *u_x_*	Displacement *u_y_*	Remark
1—Axial X	1 (*X* = 0)	0	Free	Sym *X*
	2 (*X* = *a*)	$\bar{\varepsilon_{01}}a$	Free	-
	3 (*Y* = 0)	Free	0	Sym *Y*
	4 (*Y* = *b*)	Free	Coupled	-
2—Axial Y	1 (*X* = 0)	0	Free	Sym *X*
	2 (*X* = *a*)	Coupled	Free	-
	3 (*Y* = 0)	Free	0	Sym *Y*
	4 (*Y* = *b*)	Free	$\bar{\varepsilon_{02}}b$	-
3—Shear XY	1 (*X* = 0)	Free	0	ASym *X*
	2 (*X* = *a*)	Free	0	-
	3 (*Y* = 0)	0	Free	ASym *Y*
	4 (*Y* = *b*)	$\bar{\gamma_{03}}b$	Free	-

**Table 2:** Definition of the elastic moduli and Poisson’s ration in an orthotropic material [36].

Load Case	Non-Zero Averaged Stress	Elastic Moduli	Poisson’s Ratio
1	${\bar{\sigma }}_{x}$	$E_{1}=\frac{{\bar{\sigma }}_{x}}{{\bar{\varepsilon }}_{x}}$	$\nu_{12}=-\frac{{\bar{\varepsilon }}_{y}}{{\bar{\varepsilon }}_{x}}$
2	${\bar{\sigma }}_{y}$	$E_{2}=\frac{{\bar{\sigma }}_{y}}{{\bar{\varepsilon }}_{y}}$	$\nu_{21}=-\frac{{\bar{\varepsilon }}_{x}}{{\bar{\varepsilon }}_{y}}$
3	${\bar{\tau }}_{xy}$	$G_{12}=\frac{{\bar{\tau }}_{xy}}{{\bar{\gamma }}_{xy}}$	-

**Table 3:** Comparative table of dataset influence over the training process.

Configuration	Dataset Size	Initial Training Loss	Final Training Loss	Initial Validation Loss	Final Validation Loss	Notes
4 × 4 Model	90% of solutions from brute force	0.004	0.00002	0.0017	0.00008	Loss stabilizes after approx. 60 epochs
6 × 6 Model (Baseline)	70,000 solutions (0.00015% of total)	0.004	0.00018	0.0024	0.00055	Higher validation loss due to smaller dataset
6 × 6 Model (Augmented Data—Rotation)	140,000 solutions (0.0003% of total)	0.0035	0.00016	0.002	0.0004	Validation loss reduced by 27%
6 × 6 Model (Augmented Data—Rotation & Symmetry	280,000 solutions (0.0006% of total)	0.0035	0.00015	0.002	0.00035	Further loss reduction
6 × 6 Model (Augmented Data—Rotation & Transfer Learning from 4 × 4)	140,000 solutions (0.0003% of total)	0.0022	0.00015	0.0013	0.00035	Faster initial convergence, reduced dataset requirement

## Data Availability

The raw data supporting the conclusions of this article will be made available by the authors on request.
